# BRCA1 and BRCA2 Tumor Suppressor Function in Meiosis

**DOI:** 10.3389/fcell.2021.668309

**Published:** 2021-04-23

**Authors:** Qianyan Li, JoAnne Engebrecht

**Affiliations:** Department of Molecular and Cellular Biology, and Biochemistry, Molecular, Cellular and Developmental Biology Graduate Group, University of California, Davis, Davis, CA, United States

**Keywords:** BARD1, BRCA1, BRCA2, DSBs, meiosis, MSCI, recombination

## Abstract

Meiosis is a specialized cell cycle that results in the production of haploid gametes for sexual reproduction. During meiosis, homologous chromosomes are connected by chiasmata, the physical manifestation of crossovers. Crossovers are formed by the repair of intentionally induced double strand breaks by homologous recombination and facilitate chromosome alignment on the meiotic spindle and proper chromosome segregation. While it is well established that the tumor suppressors BRCA1 and BRCA2 function in DNA repair and homologous recombination in somatic cells, the functions of BRCA1 and BRCA2 in meiosis have received less attention. Recent studies in both mice and the nematode *Caenorhabditis elegans* have provided insight into the roles of these tumor suppressors in a number of meiotic processes, revealing both conserved and organism-specific functions. BRCA1 forms an E3 ubiquitin ligase as a heterodimer with BARD1 and appears to have regulatory roles in a number of key meiotic processes. BRCA2 is a very large protein that plays an intimate role in homologous recombination. As women with no indication of cancer but carrying BRCA mutations show decreased ovarian reserve and accumulated oocyte DNA damage, studies in these systems may provide insight into why BRCA mutations impact reproductive success in addition to their established roles in cancer.

## Introduction

Homologous recombination (HR) is a high-fidelity pathway that mediates error-free repair of DNA double strand breaks (DSBs) and is essential for maintaining genome integrity. In somatic cells, DSBs can arise when DNA replication is impeded or following exposure to irradiation or other genotoxic stress. Cells deficient for HR show genomic instability including chromosome rearrangements, characteristic of most cancers (Negrini et al., 2010). In contrast to somatic cells, where DSBs pose a risk to genome integrity, during meiosis, hundreds of DSBs are purposely introduced by the topoisomerase-like protein SPO11 in early meiotic prophase and these meiotic DSBs must be accurately repaired for the production of euploid gametes (Lam and Keeney, 2014). As meiosis proceeds, meiotic DSBs are processed by DNA end resection to reveal 3′ overhangs (Garcia et al., 2011). The RAD51 recombinase as well as the meiosis-specific paralog DMC1 assemble on the resulting single strand DNA to form nucleoprotein filaments that mediate strand invasion and homology search for accurate repair (Shinohara and Shinohara, 2004). Meiotic DSB repair occurs concomitantly with the assembly of the synaptonemal complex (SC), the meiosis-specific multi-protein structure that forms between homologous chromosomes. In many organisms, SC assembly is driven by HR (Zickler and Kleckner, 2015). In the context of full length SC at the pachytene stage of meiotic prophase, a subset of recombination intermediates is processed into inter-homolog crossovers, which are essential for accurate separation of homologous chromosomes at meiosis I (Neale and Keeney, 2006; Baudat and de Massy, 2007). A large number of proteins are critical for HR, including the tumor suppressors BRCA1 and BRCA2, whose functions have been well characterized in somatic cells in the context of DNA damage and carcinogenesis. However, the roles of BRCA1 and BRCA2 during meiotic recombination have received less attention. Although the processing of DSBs by HR is similar in somatic cells and meiosis, meiotic recombination is unique in that SPO11 remains attached to the DNA end following DSB formation. Additionally, meiotic recombination occurs in the context of the SC and both sister and non-sister chromatids can serve as templates for repair. Thus, BRCA1 and BRCA2 function may be modified in meiosis to ensure accurate repair of meiotic DSBs. Studies in model organisms have provided insights into the roles of BRCA1 and BRCA2 in meiosis. This review will summarize the conserved and organism-specific meiotic functions of BRCA1 and BRCA2, focusing on recent studies in mice and *C. elegans*.

## BRCA1 in Complex With BARD1 Is an E3 Ubiquitin Ligase Critical for Genome Integrity

Breast cancer susceptibility gene 1 (BRCA1) is a tumor suppressor gene, germline mutations of which are linked to familial breast and ovarian cancers (Hall et al., 1990; Futreal et al., 1994; Godwin et al., 1994; Miki et al., 1994). More than two decades of research has implicated BRCA1 function in multiple cellular pathways, including transcriptional regulation, DNA damage signaling, cell cycle checkpoints, centrosome regulation and in the repair of DNA DSBs through HR (Moynahan et al., 1999; Xu et al., 1999; Deng, 2002, 2006; Yarden et al., 2002; Caestecker and Van de Walle, 2013; Hill et al., 2014; Hatchi et al., 2015). Of critical importance, its role in promoting HR is directly linked to maintenance of genome integrity (Roy et al., 2011; Prakash et al., 2015).

**FIGURE 1:**
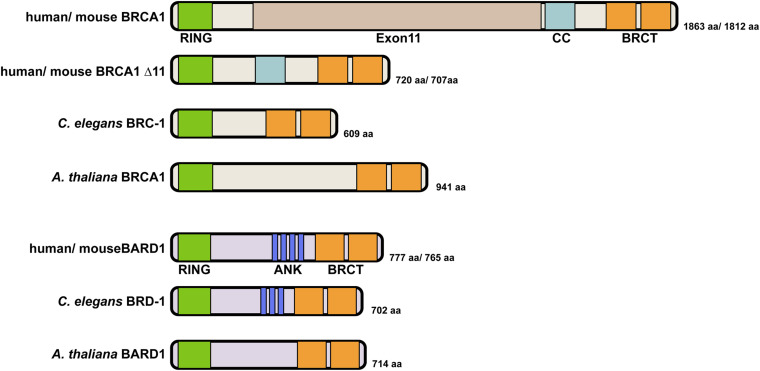
Domain structure of BRCA1 and BARD1 proteins. Human BRCA1 contains an N-terminal RING domain, an unstructured central region encoded by the large exon11 followed by a coiled coil (CC) domain and two BRCA1 C-terminal (BRCT) repeats. Both human and mouse express an alternatively spliced variant BRCA1Δ11 that contains the N-terminal RING domain and C-terminal BRCT repeats but lacks the unstructured central region (Thakur et al., 1997; Huber et al., 2001). This truncated protein is expressed in the hypomorphic *Brca1*^Δ*11*/Δ*11*^ mouse. *C. elegans* BRC-1 is structurally similar to the BRCA1Δ11 splicing variant with the presence of an N-terminal RING domain and two BRCT repeats at its C terminus. *A. thaliana* encodes a similarly structured BRCA1 ortholog that has a N-terminal RING and two C-terminal BRCT repeats. Human BARD1 and *C. elegans* BRD-1 are similar in size and domain structure, containing an N-terminal RING domain, ankyrin repeats in the middle and two C-terminal BRCT repeats. *A. thaliana* BARD-1 has a similar domain structure but appears to lack ankyrin repeats, which were not predicted by sequence alignment. BRCA1 interacts with BARD1 through their RING domains to form a heterodimer with E3 ubiquitin ligase activity.

In humans, the 1,863 amino acid BRCA1 protein has an N-terminal RING (Really Interesting New Gene) domain that coordinates two zinc cations in a cross-braced arrangement, a largely unstructured central region encoded by exon11, followed by a coiled coil domain and two C-terminal BRCT repeats (Figure 1). RING domains create a platform for binding to E2 ubiquitin conjugating enzymes and facilitate the transfer of ubiquitin from the E2 to substrates, thereby specifying E3 ubiquitin ligase activity (Deshaies and Joazeiro, 2009). The BRCT repeats are phosphopeptide interaction modules for binding to phosphorylated proteins (Manke et al., 2003; Rodriguez et al., 2003; Yu et al., 2003). BRCA1 forms a heterodimer with its obligate binding partner BARD1 (BRCA1-Associated RING Domain protein 1) through their N-terminal regions and the heterodimer exhibits efficient ubiquitin transfer activity (Wu et al., 1996; Meza et al., 1999; Brzovic et al., 2001; Hashizume et al., 2001; Baer and Ludwig, 2002). The BARD1 protein is 777 amino acids in length and similar to BRCA1, contains a RING domain at its N-terminus and two BRCT repeats at its C-terminus (Figure 1). In addition, four ankyrin repeats involved in chromatin recognition of newly replicated sister chromatids are present in the middle of the protein (Fox III, Le Trong et al., 2008; Nakamura et al., 2019). Most studies indicate that BARD1 is indispensable for BRCA1 function and depletion of BARD1 leads to highly similar phenotypes as observed for BRCA1 mutants. Mutations in BARD1 have been identified in patients with breast, ovarian and other cancer types, although at a lower frequency than BRCA1 mutations (Thai et al., 1998; Ghimenti et al., 2002). Further, as with BRCA1, loss of BARD1 results in embryonic lethality in mice as well as defects in HR leading to chromosomal instability (McCarthy et al., 2003).

The mechanisms by which BRCA1-BARD1 promotes HR during DSB repair involve multiple steps. First, BRCA1 promotes DNA end resection by antagonizing 53BP1, a DNA damage response protein that promotes error-prone non-homologous end joining (NHEJ) (Bunting et al., 2010; Daley and Sung, 2014). Two, BRCA1 regulates the MRE11-RAD50-NBS1-CtIP complex essential for DNA end processing (Cruz-Garcia et al., 2014; Aparicio et al., 2016). There is also evidence that BRCA1 removes a chromatin barrier for DNA resection through ubiquitylation of histone H2A (Densham et al., 2016). In addition to promoting resection, BRCA1-BARD1 binds to DNA and interacts with RAD51 directly, enhancing RAD51 recombinase activity by promoting homologous strand invasion and synaptic complex formation (Zhao et al., 2017). However, whether BRCA1 functions by similar mechanisms to promote HR during meiosis for the repair of SPO11-induced DSBs has remained elusive.

## BRCA1 Function in Mouse Meiosis

Mice homozygous for *Brca1* null alleles are embryonic lethal, excluding the possibility to assess BRCA1 function during meiosis (Gowen et al., 1996; Hakem et al., 1996; Liu et al., 1996; Ludwig et al., 1997). To circumvent this limitation, meiosis has been analyzed in mice carrying a hypomorphic mutation that deletes the large exon11 in the heterozygous *Trp53* (encoding p53) mutant background (*Brca1*^Δ*11*/Δ*11*^*Trp*53^+/−^) (Xu et al., 2003; Figure 1). These mice develop and survive to adulthood; lethality likely bypassed by the reduced expression of *Trp53* (Cressman et al., 1999).

### BRCA1 Is Essential for Meiotic Sex Chromosome Inactivation During Spermatogenesis

**FIGURE 2:**
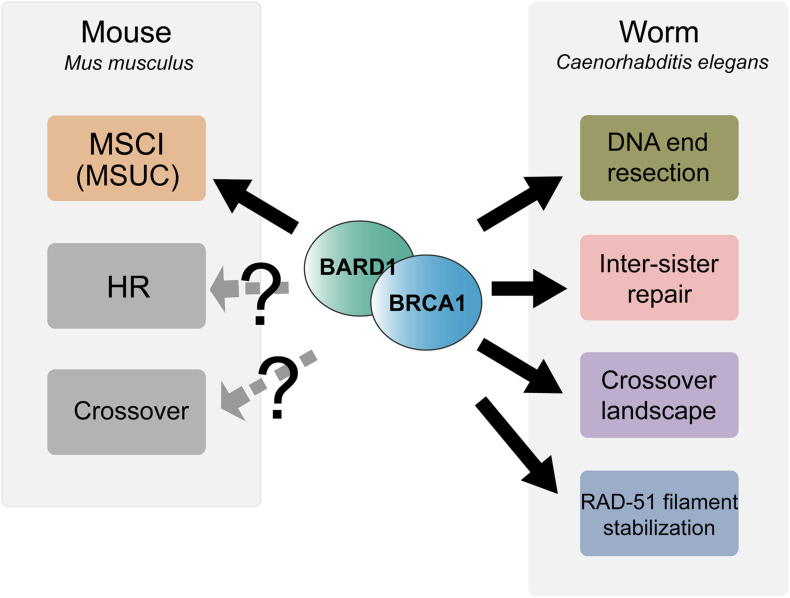
Summary of meiotic functions of BRCA1-BARD1 in mouse and *C. elegans.* BRCA1-BARD1 is critical for meiotic sex chromosome inactivation (MSCI) and meiotic silencing of unsynapsed chromatin (MUSC) during mouse meiosis while it remains an open question as to whether it functions in meiotic recombination and crossover regulation. In contrast to mouse, *C. elegans* BRC-1-BRD-1 is dispensable for MSCI but functions in DNA end resection, inter-sister recombinational repair, RAD-51 filament stabilization and regulation of the crossover landscape.

Although *Trp53* heterozygosity rescues the embryonic lethality of *Brca1*^Δ*11*/Δ*11*^ mice, males are infertile as a result of pachytene arrest and apoptotic removal of germ cells (Xu et al., 2003). This observation revealed an essential role of BRCA1 in meiotic sex chromosome inactivation (MSCI). MSCI is a repressive mechanism that occurs during meiotic prophase I and involves elaboration of heterochromatin and transcriptional silencing of non-homologous regions of sex chromosomes (Turner, 2007). MSCI is required for efficient meiotic progression in males as failure to repress the X and Y chromosomes results in elevated germline apoptosis (Figure 2).

In wild-type spermatocytes, BRCA1 localizes to asynapsed chromosome axes, including the mostly unsynapsed X and Y sex chromosomes (Scully et al., 1997). BRCA1 recruits the checkpoint kinase ataxia telangiectasia and Rad3-related protein (ATR) to the hemizygous regions of sex chromosomes; ATR phosphorylates a histone variant, H2AX, to form γH2AX, leading to sex chromosome compaction and transcriptional silencing (Fernandez-Capetillo et al., 2003; Turner et al., 2004). In the absence of full length BRCA1, ATR and γH2AX localization is disrupted, formation of XY sex body fails, and transcriptional silencing is abolished, leading to ectopic gene transcription from the hemizygous regions of the sex chromosomes (Xu et al., 2003; Turner et al., 2004; Broering et al., 2014). The inability to execute successful MSCI in the *Brca1*^Δ*11*/Δ*11*^ mutant has been attributed to a direct role of BRCA1 in establishing heterochromatin on the X and Y chromosomes and XY body morphogenesis, rather than an indirect consequence of defective meiotic recombinational repair in the absence of full-length BRCA1 (Broering et al., 2014).

The related process of meiotic silencing of unsynapsed chromatin (MSUC) also requires BRCA1 and operates in both male and female germ cells (Mahadevaiah et al., 2008; Kouznetsova et al., 2009). As with MSCI, MSUC leads to accumulation of repressive chromatin and transcriptional silencing on any asynapsed chromosomal regions. MSUC promotes the elimination of gametes with chromosome asynapsis and is initiated by the recruitment of BRCA1 to unsynapsed chromosomes through the interaction with the SC axial component SYCP3. Interestingly, oocytes have a limited capacity to silence unsynapsed chromosomes and this appears to be a consequence of the amount of BRCA1 available to accumulate on unsynapsed chromosomes. Thus, the role of BRCA1 in transcriptional silencing contributes to ensuring the production of euploid gametes.

### Potential BRCA1 Role in Meiotic Recombination

In addition to MSCI failure, spermatocytes from *Brca1*^Δ*11*/Δ*11*^ Trp53^+/–^ mice exhibited a prolonged autosomal γH2AX signal with greatly reduced numbers of RAD51 (but not DMC1) and MLH1 foci, suggesting that BRCA1 plays a role in meiotic DSBs repair and crossover formation (Xu et al., 2003). In contrast, a separate study utilizing Cre/LoxP mediated conditional germline-specific deletion of *Brca1* exon11 in the presence of both wild-type *Trp53* alleles showed that RAD51 foci were not reduced, although decreased numbers of MSH4 foci and delayed appearance of MLH1 foci were observed. These authors concluded that while BRCA1 is not essential for meiotic DSB repair, BRCA1 might be involved in the regulation of the timing of crossover formation (Broering et al., 2014). In a recent study using END-seq on mouse spermatocytes that allows direct examination of meiotic DSB processing at the single nucleotide level, hypomorphic *Brca1*^Δ*11*/Δ*11*^ Trp53^+/–^ mice did not exhibit a reduction in resection track length, suggesting that BRCA1 does not promote DNA resection in meiotic DSB repair as in somatic cells (Paiano et al., 2020). Together these results suggest that the critical meiotic role for BRCA1 is in transcriptional silencing; however, it is possible that BRCA1 function in meiotic recombination is obscured by the use of the hypomorphic *Brca1*^Δ*11*/Δ*11*^ mutant (Figure 2).

Analysis of female meiosis in the hypomorphic *Brca1*^Δ*11*/Δ*11*^ mutation revealed no observable phenotypes. Female *Brca1* mutants are fertile and the number of MLH1 foci are comparable to that observed in wild-type oogenesis, suggesting that the region deleted in *Brca1*^Δ*11*/Δ*11*^ is not required for meiotic recombination during female meiosis (Xu et al., 2003; Broering et al., 2014). Therefore, the observed sex-specific phenotypes in the hypomorphic *Brca1*^Δ*11*/Δ*11*^ mutant are likely a consequence of the presence of unsynapsed sex chromosomes in males. It is also important to note that the region encoded by exon11 is thought to be unstructured with no resemblance to known domain structures (Li and Greenberg, 2012). Future studies focusing on the RING domain, which confers E3 ubiquitin ligase activity, and the BRCT repeats, are necessary to reveal whether these domains play important roles in the repair of meiotic DSBs in both male and female meiosis. Finally, to the best of our knowledge a functional role of BARD1 in mice gametogenesis has not been examined.

## BRCA1 Function in *C. Elegans* Meiosis

### The *C. elegans* Germ Line as a Model for Studying Meiosis and BRCA1-BARD1 Function

**FIGURE 3:**
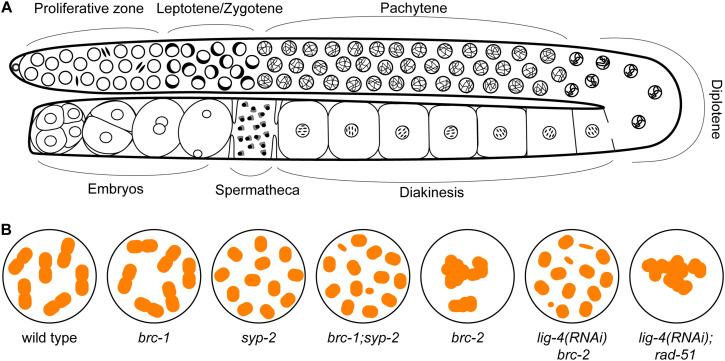
The *C. elegans* germ line presents a spatial temporal organization of events during meiotic prophase I. **(A)** At the distal proliferative zone, germline stem cells mitotically divide to produce cells that will advance down the gonad and enter meiosis. Chromosome pairing and DSBs induction by SPO-11 occur in leptotene/zygotene (transition zone), which is characterized by the presence of clustered chromatin on one side of the nuclei. DSBs are processed and bound by RAD-51 for homologous recombinational repair, which are visible as RAD-51 foci by immunostaining starting in leptotene through pachytene. The synaptonemal complex (SC) is fully assembled between the homologs in pachytene and strictly one crossover forms between each chromosome pair in late pachytene. Upon crossover formation, the SC disassembles and homologs undergo remodeling and compaction to reveal six bivalents at diakinesis stage. **(B)** Cartoon of chromosome structure observed in diakinesis nuclei in WT (6 bivalents), *brc-1* (6 bivalents), *syp-2* (12 univalents), *brc1; syp-2* (> 12 univalents/DNA fragments) (Adamo et al., 2008), *brc-2* (aggregation), *lig-4* (RNAi) *brc-2* (12 univalents with some DNA fragments) and *lig-4* (RNAi); *rad-51* (aggregation) (Martin et al., 2005) mutants.

*Caenorhabditis elegans* has emerged as an excellent model for investigating meiosis: many genes required for meiotic recombination are conserved in this metazoan and the animals possess prominent gonads that exhibit a spatial temporal organization of germ cells undergoing meiotic prophase I (Figure 3A). At the distal tip, germline stem cells divide to produce cells that will advance down the gonad and enter meiosis. In transition zone (corresponding to leptotene/zygotene), homologous chromosomes are paired together, facilitated by Zn-finger ZIM-1/2/3 and HIM-8 proteins that bind to special sequences present on each homolog pair. Beginning at this stage, SPO-11 induces meiotic DSBs, which are processed and bound by RAD-51 for homologous recombinational repair. In pachytene, the SC is fully assembled between the homologs and within this context strictly one crossover forms between each chromosome pair in late pachytene. Upon crossover formation, the SC disassembles and homologs undergo remodeling and compaction to reveal six bivalents at diakinesis stage, representing the six pairs of homologs connected by chiasmata (Figure 3B; Lui and Colaiacovo, 2013; Hillers et al., 2017). Although the overall process is very similar to other systems, it is important to note that there are differences unique to *C. elegans* meiosis. These include the absence of DMC1 in this organism, thus RAD-51 is the sole recombinase acting during both mitotic and meiotic recombination (Brown and Bishop, 2014). Interestingly, *C. elegans* RAD-51 contains three amino acids conserved in the DMC1 lineage that stabilize mismatch-containing heteroduplex DNA, critical for meiotic recombinase function (Steinfeld et al., 2019). Another unique feature of *C. elegans* meiosis is that chromosome synapsis does not depend on meiotic recombination initiation (Dernburg et al., 1998). Nevertheless, the availability of molecular markers combined with genetic and genomic approaches has made the *C. elegans* germ line a powerful system that provides a unique opportunity to dissect gene function at any particular sub-stage of meiotic prophase. Most importantly, proteins with conserved domain structure and sequence similarity to BRCA1 and BARD1, referred to as BRC-1 and BRD-1, are encoded in the *C. elegans* genome. *brc-1* and *brd-1* null mutants exhibit elevated IR sensitivity and a higher incidence of males among self-progeny (a readout of X chromosome non-disjunction) compared to wild type, but are mostly fertile, allowing analysis of meiotic outcomes in the absence of functional BRCA1 and BARD1 (Boulton et al., 2004; Li et al., 2018). Similar to *C. elegans*, *Arabidopsis AtBRCA1* and *AtBARD1* mutants are also fertile, suggesting that the essentiality of mammalian BRCA1-BARD1 is not broadly conserved (Reidt et al., 2006).

*C. elegans brc-1* encodes a 609 amino acid protein with highly conserved N-terminal RING domain and C terminal BRCT repeats, similar to the human protein. Structurally, *C. elegans* BRC-1 is analogous to the BRCA1Δ11 splicing variant (Figure 1). AtBRCA1 with 941 amino acids is also considerably smaller than the human protein. The *C. elegans* BRD-1 and AtBARD1 proteins are similar in both size and domain architecture to the human protein, although AtBARD1 does not have recognizable ankyrin repeats (Figure 1). Interestingly, *C. elegans* BRC-1-BRD-1 exhibits dynamic localization throughout meiotic prophase. Discrete foci of BRC-1-BRD-1 that partially colocalize with RAD-51 are present in both proliferative/mitotic region and early meiotic prophase, from leptotene to early pachytene (Li et al., 2018, 2020). As meiotic prophase progresses, BRC-1-BRD-1 localizes with the SC between the maternal and paternal chromosomes (Polanowska et al., 2006; Janisiw et al., 2018; Li et al., 2018). This localization is in contrast to BRCA1 localization in mammalian meiocytes, where BRCA1 is found on the axes of asynapsed chromosomes (Turner et al., 2004). In late pachytene upon crossover maturation, BRC-1-BRD-1 concentrates on one subdomain of the chromosome pair termed the “short arm”, suggesting an intimate connection of BRC-1-BRD-1 to crossover sites and potential involvement in crossover regulation.

### BRC-1-BRD-1 Is Not Essential for Meiotic Sex Chromosome Inactivation but Promotes HR in Spermatogenesis

*C. elegans* BRC-1-BRD-1 is absent from the single asynapsed X chromosome in male germ cells, and consistent with this observation, BRC-1-BRD-1 is not required for MSCI during spermatogenesis. In *brc-1* and *brd-1* null mutants, deposition of the repressive chromatin mark H3K9me2 and the absence of Pol2-S2P (actively transcribing RNA polymerase II) signal on the X chromosome are indistinguishable from wild-type animals, suggesting that MSCI is successful in these mutants. As such, the null mutants do not exhibit pachytene arrest and germ cells complete meiotic prophase in preparation for the meiotic divisions (Li et al., 2020).

Analysis of RAD-51 immunostaining in the *brc-1* and *brd-1* null male germ lines showed reduced levels of RAD-51 foci in early meiotic prophase and this reduction was suppressed by inhibiting the NHEJ pathway. Moreover, quantification of GFP:RPA-1 foci, indicative of single stranded DNA, showed a significant reduction in overall foci number and intensity in the absence of BRC-1-BRD-1, suggesting that BRC-1-BRD-1 favors HR at the expense of NHEJ through promoting resection of DSBs during male meiosis (Li et al., 2020; Figure 2). This role is similar to what is proposed for BRCA1 function in promoting HR in somatic cells.

### BRC-1-BRD-1 Promotes Inter-Sister Recombination and Stabilizes the RAD-51 Filament Under Checkpoint Activation in Oogenesis

In contrast to male meiosis, *brc-1* and *brd-1* null mutants exhibited an increased number of RAD-51 foci at late pachytene in oogenic germ lines, with no obvious difference in RAD-51 kinetics in early meiotic prophase as compared to wild-type animals (Adamo et al., 2008; Janisiw et al., 2018; Li et al., 2018). The elevated RAD-51 foci observed in late pachytene suggests that the repair of a subset of DSBs is delayed in the absence of BRC-1-BRD-1. The high fertility and presence of six bivalents, representing the six homologs connected by chiasmata, at diakinesis in *brc-1* and *brd-1* mutants (Figure 3B) suggest that BRC-1-BRD-1 is not essential for crossover formation. To test the hypothesis that BRC-1 promotes repair of DSBs by the inter-sister recombination pathway, Adamo and coworkers disrupted SC assembly and thereby inter-homolog crossovers by mutation of *syp-2* (one of six components in the central region of the SC) in the *brc-1* mutant. *syp-2* mutants have twelve intact univalents at diakinesis (Figure 3B), suggesting efficient repair of DSBs by the inter-sister pathway. On the other hand, in the *brc-1; syp-2* double mutant more than twelve DAPI staining bodies were often observed (Figure 3B), indicating the presence of chromosome fragmentation and failure in inter-sister repair. These results are consistent with BRC-1 playing an important role in inter-sister repair during oogenesis (Adamo et al., 2008). A recent study extended these findings by showing that mutation of *brc-1* enhanced the phenotype of phosphorylation defective mutants in *syp-1* (another component of the central region of the SC), presumably through impairment of inter-sister recombination (Garcia-Muse et al., 2019; Figure 2). Importantly, BRC-1-dependent inter-sister repair prevents erroneous recombination (recombination between heterologous sequences) in meiosis, suggesting one mechanism by which BRC-1 prevents genome instability (Leon-Ortiz et al., 2018).

In addition to promoting inter-sister repair, BRC-1 is required to stabilize the RAD-51 filament from premature disassembly in late pachytene under meiotic checkpoint activation conditions. In *zim-1/2/3* or *syp-1* mutants, which lack crossovers on a subset or all chromosomes, respectively, and activate meiotic checkpoints, extensive RAD-51 foci are present throughout meiotic prophase (Yu et al., 2016). Removing BRC-1 in these mutant backgrounds results in a region in late pachytene with significantly reduced RAD-51 levels, with high levels of RAD-51 both prior to and after this region. Both the number of RAD-51 foci as well as the fluorescence intensity of residual foci was greatly diminished in this region and thus this pattern has been referred to as a RAD-51 “dark zone”. Taking advantage of the spatial temporal organization of the germ line, time course analysis of *spo-11; brc-1; syp-1* mutants exposed to irradiation (IR) was performed. The *spo-11* mutant was used so that breaks could be induced uniformly in the germ line at a single point in time by IR and as nuclei moved through the germ line no new breaks were formed. This analysis revealed that RAD-51 installed on processed DSBs in nuclei residing in early prophase at the time of DSB induction was dismantled once the nuclei reached late pachytene, suggesting that BRC-1 promotes the stability of the RAD-51 filament under these conditions (Li et al., 2018). The mechanism underlying BRC-1-dependent RAD-51 stabilization is currently unknown and could be either through direct interaction with RAD-51 to reduce its ATP hydrolysis and/or regulation of helicases which dismantle the RAD-51 filament. Interestingly, the requirement for BRC-1 to stabilize RAD-51 filaments under checkpoint activation conditions is oogenesis-specific, as a RAD-51 dark zone was not observed in the male germ line (Li et al., 2020; Figure 2).

Recent studies examining the mutational signatures of *brc-1* and *brd-1* mutants propagated over multiple generations revealed elevated levels of small deletions, deletions-insertions, single nucleotide variants and tandem repeats (Kamp et al., 2020; Volkova et al., 2020). Analysis of *brc-1* and *brd-1* mutants in combination with mutations in different repair pathways provided evidence that theta-mediated end joining (TMEJ), but not NHEJ, was responsible for the mutational profiles observed. TMEJ anneals short regions of microhomology and catalyzes template-dependent DNA synthesis to repair the broken DNA molecule. These results suggest that in the absence of BRC-1-BRD-1, TMEJ repairs inefficiently resected DSBs. It will be important to distinguish whether the mutations are a consequence of repair of meiotic DSBs, or repair of breaks generated during replication prior to meiotic entry or during embryogenesis, to understand the complete spectrum of BRC-1-BRD-1 function in both the soma and in meiosis. Nonetheless, the mutational profile of *C. elegans brc-1* and *brd-1* mutants is very similar to that found in BRCA1-deficient tumor cells, suggesting that TMEJ repair in the absence of BRCA1 contributes to carcinogenesis (Kamp et al., 2020; Volkova et al., 2020).

### BRC-1-BRD-1 Regulates Crossover Patterning

Given that there are many more DSBs than crossovers, a subset of processed DSBs is chosen to be resolved as crossovers in a process referred to as crossover designation (Gray and Cohen, 2016). To investigate whether BRC-1 plays a role in crossover designation and/or resolution, genetic linkage analysis on meiotic products of *brc-1* mutants was performed and revealed an altered crossover landscape. Although the genetic map length was not significantly different between wild type and *brc-1* mutants, there was a shift in crossover distribution from chromosome arms, which are most often observed in wild-type animals, to more central regions on chromosomes (Li et al., 2018, 2020). Altered crossover distribution to the chromosome center has been observed in many other *C. elegans* mutants defective for various aspects of meiotic recombination (Zetka and Rose, 1995; Wagner et al., 2010; Meneely et al., 2012; Saito et al., 2012, 2013; Chung et al., 2015; Hong et al., 2016; Jagut et al., 2016). While the underlying mechanisms are currently unknown, it has been suggested that this could result from an altered chromatin landscape (Saito and Colaiacovo, 2017). Thus, BRCA1 may regulate chromatin structure in *C. elegans* meiosis, as it does in mouse meiosis (Broering et al., 2014; Densham et al., 2016), although the specific types of chromatin modification regulated by BRCA1 may not be identical in *C. elegans* and mouse.

Surprisingly, in the *zim-1* mutant where two chromosomes fail to pair and synapse, BRC-1-BRD-1 promoted the formation of extra COSA-1 marked crossover designation events on the remaining chromosome pairs during oogenesis. COSA-1 (CrossOver Site Associated protein 1) is generally accepted to mark canonical crossovers in *C. elegans* meiosis (Yokoo et al., 2012); therefore, the number of COSA-1 foci has been used as a cytological readout of the number of genetic crossovers. The reduced COSA-1 foci in the *brc-1; zim-1* double mutant, however, was not accompanied by a smaller genetic map distance, measured by SNP marker-based linkage analysis. These results suggest that not all crossovers are marked by COSA-1 in the *brc-1; zim-1*double mutant. Further, while the map length was similar in the absence of BRC-1, CO patterning was altered such that there were elevated levels of single crossovers (SCOs) with a concomitant reduction in double crossovers (DCOs). As a crossover can form between any two non-sister chromatids within paired homologs, two, three or four-strand DCOs are possible outcomes of elevated crossover formation. However, only DCOs between the same two chromatids can be detected as DCOs in SNP marker-based analysis, because only one sister chromatid is inherited in the product of meiosis. DCOs involving three or four chromatids will be detected as SCOs. Therefore, the aforementioned observation is consistent with a model whereby inactivation of BRC-1 in the *zim-1* mutant results in a shift from two-strand DCOs that are marked by COSA-1 and observed in the DCO class, to three- and four-strand DCOs that lack the COSA-1 marker and are detected as SCOs (Li et al., 2018). In contrast to oogenesis, BRC-1 inhibits the formation of extra COSA-1 marked crossover precursors in spermatogenesis. Elevated levels of COSA-1 foci were observed in the *brc-1; zim-1* double mutant as compared to *zim-1*. Additionally, the genetic map distance was enlarged in the *brc-1*; *zim-1* double mutant, suggesting that BRC-1 inhibits the formation of extra canonical crossovers in spermatogenesis (Li et al., 2020). Together, these results suggest that BRC-1 plays a role in CO patterning, perhaps through regulating both canonical and non-canonical CO pathways under conditions of meiotic dysfunction (Figure 2).

Why does *brc-1* and *brd-1* mutation exhibit sex-specific phenotypes? One hypothesis is that BRC-1-BRD-1 interacts with unique partners to form different complexes during male and female meiosis. This would be analogous to what has been established for BRCA1 function in somatic cells, where it forms three different complexes with distinct functions under different physiological conditions (Li and Greenberg, 2012). Alternatively, or in addition, the sex-specific phenotypes could be a consequence of BRC-1-BRD-1 being differentially regulated by post-translational modifications in the diverging environments of male and female meiosis. Future studies on BRC-1-BRD-1 interacting proteins and the regulation of complex(es) will provide insight into the functions of BRC-1-BRD-1 during spermatogenesis and oogenesis. These studies may also shed light on the sex-specific regulation of the BRCA1-BARD1 complex in mammals.

## BRCA2 Functions as an Essential Mediator for HR

Breast cancer susceptibility gene 2 (BRCA2) is an essential mediator of HR (Jensen et al., 2010; Liu et al., 2010; Kowalczykowski, 2015). Similar to *BRCA1*, germline mutations in *BRCA2* predispose patients to breast and ovarian cancer and genome instability (Wooster et al., 1995; Yu et al., 2000; Venkitaraman, 2002; King et al., 2003). Biochemical, cell biological and genetic studies have supported a role of BRCA2 in recruiting the RAD51 recombinase to resected single strand DNA at DSBs and promoting nucleoprotein filament assembly to mediate homology search and strand exchange (Sharan et al., 1997; Wong et al., 1997; Abbott et al., 1998; Chen et al., 1999; Tutt et al., 1999; Yuan et al., 1999; Moynahan et al., 2001; Xu et al., 2001; Jensen et al., 2010; Liu et al., 2010; Thorslund et al., 2010).

**FIGURE 4:**
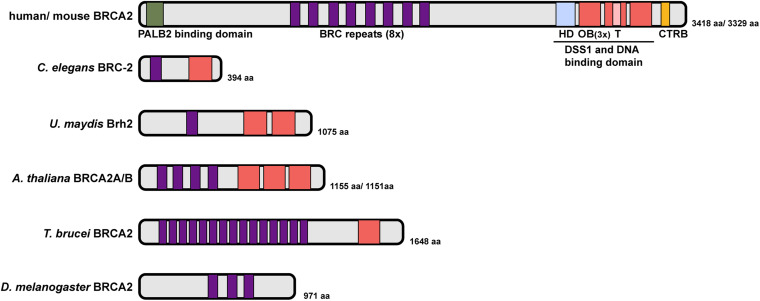
Domain structure of BRCA2 proteins. Human BRCA2 encodes an exceptionally large protein with an N-terminal PALB2 binding domain, eight BRC repeats, a DSS1 and DNA binding domain (DBD) composed of one helix-rich domain (HD), three oligonucleotide/oligosaccharide binding (OB) folds and a tower domain, and a C terminal RAD51 binding domain (CTRB). *C. elegans* BRC-2 represents a simplified version with a single BRC repeat and OB fold. The number of BRC repeats and OB fold domains vary greatly in different organisms (*U. maydis* Brh2, *A. thaliana* BRCA2A/B, *T. brucei* BRCA2 and *D. melanogaster* BRCA2). Sequence alignment did not identify a putative OB fold/DNA binding domain in *Drosophila* BRCA2 (Yang et al., 2002).

Human BRCA2 encodes an exceptionally large protein consisting of 3,418 amino acids with multiple functional domains: an N-terminal domain that facilitates binding with Partner And Localizer of BRCA2 (PALB2), eight BRC repeats that define the RAD51 binding motif, a DSS1 and DNA binding domain (DBD, composed of one helix-rich domain (HD), three oligonucleotide/oligosaccharide binding (OB) folds and a tower domain), and a C terminal RAD51 binding domain (CTRB) (Figure 4; Yang et al., 2002; Esashi et al., 2005; Xia et al., 2006; Carreira et al., 2009; Shivji et al., 2009). Given its essential role in HR, it is not surprising that BRCA2 is conserved in fungi, plants and metazoans. While overall similar, BRCA2 orthologs possess different numbers of BRC repeats and OB folds, which are signature domains of BRCA2, and vary considerably in size (Gudmundsdottir and Ashworth, 2004; Figure 4). For example, Brh2, the BRCA2 ortholog in the fungus *Ustilago maydis*, contains a single BRC repeat and two OB folds (Kojic et al., 2002, 2005). *Drosophila melanogaster* BRCA2 contains three BRC repeats but no recognizable OB fold (Klovstad et al., 2008). Two almost identical BRCA2 orthologs were identified in *Arabidopsis thaliana*, each containing four BRC repeats (Siaud et al., 2004). In contrast, the parasite *Trypanosoma brucei* possess a single BRCA2 ortholog with 15 BRC repeats (Hartley and McCulloch, 2008). The BRC repeat is highly conserved among species; despite the different number of repeats, BRC domains in all BRCA2 orthologs examined so far have been shown to bind RAD51 directly and to promote RAD51 nucleoprotein filament formation on ssDNA, which is essential for homology search and strand exchange during HR. In addition, BRCA2 interaction with the highly conserved DSS1 protein also contributes to HR through promoting RAD51-recruitment activity and stability of BRCA2 (Li et al., 2006; Liu et al., 2010; Siaud et al., 2011). The CTRB domain, while conferring RAD51 binding and stabilizing RAD51 filaments on ssDNA, is not essential for HR (Davies and Pellegrini, 2007; Esashi et al., 2007; Prakash et al., 2015).

*C. elegans* BRCA2 (BRC-2) contains domain signatures similar to mammalian BRCA2 but is approximately 1/8 the size, with just 394 amino acids. BRC-2 contains a single BRC repeat that directly interacts with RAD51 and a single OB fold that preferentially binds to ssDNA (Martin et al., 2005; Petalcorin et al., 2006; Figure 4). The single BRC repeat is comprised of two RAD-51 interaction regions, one that preferentially binds to free RAD-51, and the other to the RAD-51-DNA nucleoprotein filament that exhibits inhibitory activity on RAD-51 ATPase hydrolysis. Together, these two RAD-51 interaction regions within the BRC repeat are proposed to coordinate the activity of BRC-2 for promoting RAD-51 nucleation on ssDNA and stabilizing existing RAD51 filament from disassembly through inhibiting ATP hydrolysis (Petalcorin et al., 2007). Recent single-molecule analysis has revealed that BRC-2 acts primarily as a RAD-51 nucleation factor on RPA-coated ssDNA (Belan et al., 2021).

### BRCA2 Role in Meiotic Recombination

**FIGURE 5:**
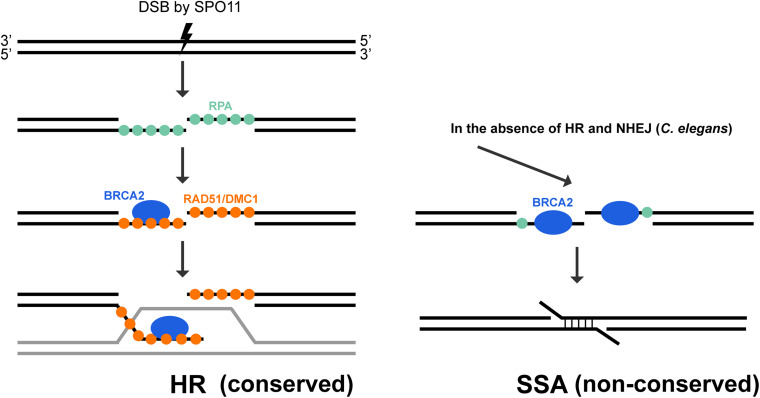
Conserved and non-conserved roles of BRCA2 during meiosis. BRCA2 is an essential mediator of homologous recombination in meiosis. After SPO-11 induced DSB is resected, the 3′ ssDNA is coated with RPA. BRCA2 is critical for recruiting DMC1/RAD51 recombinases to displace RPA molecules on the ssDNA, promoting the formation and stabilization of nucleoprotein filaments to mediate homology search and strand exchange. This function of BRCA2 is highly conserved during meiosis among a large variety of organisms, including *C. elegans*. However, *C. elegans* BRC-2 also exhibits a non-conserved role in promoting single strand annealing when HR (*rad-51* mutant) and NHEJ (*lig-4* knock down) are not available for repair (Martin et al., 2005).

In addition to a role of promoting RAD51 mediated HR in somatic cells, studies on BRCA2 orthologs have revealed a requirement for BRCA2 during meiosis. In *Ustilago maydis*, mutation of Brh2 led to a failure in the formation of meiotic spore products (Kojic et al., 2002). Null mutants of BRCA2 ortholog in *Drosophila* led to sterility in both male and female flies (Klovstad et al., 2008; Weinberg-Shukron et al., 2018). A transgenic mouse line expressing low levels of human BRCA2 in the gonad showed reduced RAD51 and DMC1 foci formation and prophase arrest of spermatocytes, due to the inability to complete meiotic recombination (Sharan et al., 2004). Depletion of *A. thaliana* BRCA2 by RNAi showed meiotic defects similar to *rad51; dmc1* double mutants (Siaud et al., 2004) and *C. elegans brc-2* mutant produced completely inviable progeny (Martin et al., 2005), suggesting an indispensable role of BRCA2 during meiosis. Studies on human and *Arabidopsis* BRCA2 proteins demonstrated that BRCA2 directly binds to the meiosis-specific recombinase DMC1, which functions together with RAD51 to promote strand invasion and joint molecule formation during meiotic recombination (Dray et al., 2006; Thorslund et al., 2007; Jensen et al., 2010; Martinez et al., 2016). As with RAD51, the BRC repeats facilitate binding between BRCA2 and DMC1, although binding affinities for each individual BRC repeat differ between RAD51 and DMC1 (Martinez et al., 2016). Moreover, different mechanisms have been proposed for BRCA2 stimulation of RAD51 versus DMC1 recombinase activity. In the context of RAD51 mediated recombination, BRCA2 and its eight BRC repeats function by a combination of inhibiting RAD51 ATPase activity, promoting RAD51 filament formation on ssDNA but not dsDNA, and enhancing strand exchange activity of RAD51. In contrast, stabilization of DMC1 filament on ssDNA was proposed to be the major mechanism by which BRCA2 functions with DMC1 (Martinez et al., 2016; Figure 5).

BRCA2 localization to DSBs in somatic cells depends on PALB2 (Xia et al., 2006). It has remained mysterious until recently, how BRCA2 is recruited to DSBs during meiosis. The Shibuya group identified a BRCA2 localizer in mice, which they named meiotic localizer of BRCA2 (MEILB2). MEILB2 is specifically expressed in germ cells and localizes to meiotic recombination sites on the chromosome axis. In the absence of MEILB2, the recruitment of DMC1 and RAD51 recombinase to meiotic DSBs is abolished, leading to sterility in male mice. Furthermore, MEILB2 directly binds to BRCA2 *in vitro* and is a physiological binding partner of BRCA2 *in vivo*. Removing MEILB2 impairs BRCA2 localization to resected ssDNA in spermatocytes, suggesting that MEILB2 recruits BRCA2 to sites undergoing meiotic recombination (Zhang et al., 2019). In contrast to males, female *Meilb2^–/–^* mice show only a ∼50% reduction in the localization of DMC1 and RAD51, and are sub-fertile, suggesting that redundant mechanisms exist to localize BRAC2 in oogenesis. One possibility is that PALB2 functions in concert with MEILB2 in female meiosis to localize BRCA2. Interestingly, PALB2 knockout mice show reduced male, but not female, fertility. This reduction in fertility is likely due to PALB2 interaction with BRCA1 (Simhadri et al., 2014). Future studies addressing the roles, redundancies and interconnections between PALB2, BRCA1 and BRCA2 will be important for understanding how meiotic DSBs are processed in male and female meiosis. Recently a third component of the BRCA2 complex, BRCA2 and MEILB2-associating protein 1 (BRME1), was identified. BRME1 forms a ternary complex with BRCA2 and MEILB2 and in the absence of BRME1, meiotic DSB repair, homologous chromosome synapsis and crossover formation were impaired in spermatogenesis (Takemoto et al., 2020; Zhang et al., 2020). MEILB2 is conserved among vertebrate taxa; whether binding partners promote meiotic regulation of BRCA2 in organisms such as worms and plants remain to be investigated.

### Non-conserved Role of BRCA2 in *C. elegans* Meiosis

BRCA2’s role in promoting RAD51/DMC1 nucleoprotein filament formation for homology search and strand exchange in meiotic recombination is conserved among all organisms where it has been examined. A RAD-51 independent, non-conserved role of BRC-2 was uncovered in *C. elegans* meiosis (Martin et al., 2005; Petalcorin et al., 2006). Without BRC-2, SPO-11 induced DSBs are resected, but RAD-51 is not recruited to the single stranded DNA, blocking strand invasion for error-free repair. As the presence of DSBs is extremely deleterious, alternative repair pathways are engaged to remove any remaining breaks before cells exit meiotic prophase I. In *rad-51* or *brc-2* single mutant, oogenic diakinesis nuclei exhibit aggregated DAPI staining chromosome structures, in contrast to the six morphologically distinct bivalent structures in wild-type animals (Figure 3B). Inactivating NHEJ (*lig-4*) in the *brc-2* mutant resulted in mostly twelve DAPI bodies (Figure 3B), suggesting that the aggregation observed in *brc-2* is due to inappropriate repair of meiotic DSBs by NHEJ. However, when a functional BRC-2 was present, as in the case of the *lig-4; rad-51* double mutant, diakinesis nuclei contained clumped DAPI structures as seen in *brc-2* and *rad-51* single mutants (Figure 3B; Martin et al., 2005). This observation suggests that BRC-2 promotes an alternative repair pathway when both HR and NHEJ fail to be executed in meiocytes. A possible candidate for this repair pathway is single strand annealing (SSA). Indeed, *in vitro* experiments showed that purified *C. elegans* BRC-2 protein promoted annealing of single strand oligonucleotide coated with RPA (Petalcorin et al., 2006), an activity that mammalian BRCA2 does not possess (Jensen et al., 2010; Figure 5). It is likely that *C. elegans* BRC-2 has acquired this function to promote SSA during meiosis, as an ortholog of RAD52, which mediates SSA, is missing.

## Conclusion

That organisms such as mice, *C. elegans*, and *A. thaliana* carrying mutations in their respective BRCA1 and BRCA2 orthologs exhibit meiotic phenotypes is consistent with BRCA1 and BRCA2 playing critical roles in meiosis. While important for meiotic recombination, BRCA1 and BRCA2 orthologs have acquired divergent functions throughout evolution. BRCA1 together with BARD1 functions as an E3 ubiquitin ligase that promotes ubiquitin transfer to a number of substrates and therefore plays regulatory roles in various processes. Not surprisingly, BRCA1 function during meiosis is quite diverse in different organisms (Figure 2). For example, BRCA1 is essential for MSCI in mice but is dispensable for MSCI in *C. elegans*, while *C. elegans* BRC-1 promotes DNA end resection, stabilizes the RAD-51 filament and regulates the crossover landscape. It remains an open question whether BRCA1-BARD1 functions in any of these aspects of meiotic recombination in mammals. Future studies taking advantage of conditional expression and genome editing tools should facilitate analyses on the role of E3 ligase activity, including identification of substrates, and the conserved BRCT domains. In contrast to BRCA1, BRCA2 plays a fundamental and conserved role in HR as a mediator to recruit RAD51 and DMC1 for nucleoprotein filament formation and strand invasion. However, *C. elegans* BRC-2 also uniquely promotes the alternative SSA pathway, perhaps as a consequence of a streamlined set of repair proteins (e.g., absence of DMC1 and RAD52) (Figure 5). While not identical, knowledge on meiotic roles of BRCA1 and BRCA2 from model organisms will continue to provide valuable insights into the mechanisms by which these two genes function during human meiosis. Clinical data has shown a correlation between the presence of BRCA1 and BRCA2 mutations in healthy carriers and ovarian aging, which is measured by elevated accumulation of DNA damage in oocytes and reduced primordial follicle reserve (Oktay et al., 2010; Lin et al., 2017; Lambertini et al., 2018). This indicates that the functions of BRCA1 and BRCA2 during human meiosis are likely to influence sperm and egg quality. Interestingly, some cancers inappropriately express meiotic genes and recent evidence suggests that this may lead to altered BRCA2 function (Hosoya et al., 2011; Zhang et al., 2020). HR was inhibited in somatic cells when the SC protein SYCP3 and the meiotic partners of BRCA2, MEILB2 and BRME1, were aberrantly expressed, presumably as a result of BRCA2 protein being sequestered when bound by the meiotic proteins. Future studies focusing on meiotic aspects of BRCA1 and BRCA2 may advance our knowledge in human reproduction as well as tumorigenesis to provide tools for improving fertility and health.

## Author Contributions

QL wrote the manuscript with content and editorial input from JE. Both authors contributed to the article and approved the submitted version.

## Conflict of Interest

The authors declare that the research was conducted in the absence of any commercial or financial relationships that could be construed as a potential conflict of interest.

## References

[B1] Abbott D. W. Freeman M. L. Holt J. T. (1998). Double-strand break repair deficiency and radiation sensitivity in BRCA2 mutant cancer cells. *J. Natl. Cancer Inst.* 90 978–985. 10.1093/jnci/90.13.978 9665145

[B2] Adamo A. Montemauri P. Silva N. Ward J. D. Boulton S. J. La Volpe A. (2008). BRC-1 acts in the inter-sister pathway of meiotic double-strand break repair. *EMBO Rep.* 9 287–292. 10.1038/sj.embor.7401167 18219312 PMC2267377

[B3] Aparicio T. Baer R. Gottesman M. Gautier J. (2016). MRN, CtIP, and BRCA1 mediate repair of topoisomerase II-DNA adducts. *J. Cell Biol.* 212 399–408. 10.1083/jcb.201504005 26880199 PMC4754713

[B4] Baer R. Ludwig T. (2002). The BRCA1/BARD1 heterodimer, a tumor suppressor complex with ubiquitin E3 ligase activity. *Curr. Opin. Genet. Dev.* 12 86–91. 10.1016/s0959-437x(01)00269-6 11790560

[B5] Baudat F. de Massy B. (2007). Regulating double-stranded DNA break repair towards crossover or non-crossover during mammalian meiosis. *Chromosome Res.* 15 565–577. 10.1007/s10577-007-1140-3 17674146

[B6] Belan O. Barroso C. Kaczmarczyk A. Anand R. Federico S. O’reilly N. (2021). Single-molecule analysis reveals cooperative stimulation of Rad51 filament nucleation and growth by mediator proteins. *Mol. Cell* 81 1058.e7–1073.e7. 33421363 10.1016/j.molcel.2020.12.020 PMC7941204

[B7] Boulton S. J. Martin J. S. Polanowska J. Hill D. E. Gartner A. Vidal M. (2004). BRCA1/BARD1 orthologs required for DNA repair in *Caenorhabditis elegans*. *Curr. Biol.* 14 33–39. 10.1016/j.cub.2003.11.029 14711411

[B8] Broering T. J. Alavattam K. G. Sadreyev R. I. Ichijima Y. Kato Y. Hasegawa K. (2014). BRCA1 establishes DNA damage signaling and pericentric heterochromatin of the X chromosome in male meiosis. *J. Cell Biol.* 205 663–675. 10.1083/jcb.201311050 24914237 PMC4050732

[B9] Brown M. S. Bishop D. K. (2014). DNA strand exchange and RecA homologs in meiosis. *Cold Spring Harb. Perspect. Biol.* 7:a016659. 10.1101/cshperspect.a016659 25475089 PMC4292170

[B10] Brzovic P. S. Rajagopal P. Hoyt D. W. King M. C. Klevit R. E. (2001). Structure of a BRCA1-BARD1 heterodimeric RING-RING complex. *Nat. Struct. Biol.* 8 833–837. 11573085 10.1038/nsb1001-833

[B11] Bunting S. F. Callen E. Wong N. Chen H. T. Polato F. Gunn A. (2010). 53BP1 inhibits homologous recombination in Brca1-deficient cells by blocking resection of DNA breaks. *Cell* 141 243–254. 10.1016/j.cell.2010.03.012 20362325 PMC2857570

[B12] Caestecker K. W. Van de Walle G. R. (2013). The role of BRCA1 in DNA double-strand repair: past and present. *Exp. Cell Res.* 319 575–587. 10.1016/j.yexcr.2012.11.013 23200932

[B13] Carreira A. Hilario J. Amitani I. Baskin R. J. Shivji M. K. Venkitaraman A. R. (2009). The BRC repeats of BRCA2 modulate the DNA-binding selectivity of RAD51. *Cell* 136 1032–1043. 10.1016/j.cell.2009.02.019 19303847 PMC2669112

[B14] Chen C. F. Chen P. L. Zhong Q. Sharp Z. D. Lee W. H. (1999). Expression of BRC repeats in breast cancer cells disrupts the BRCA2-Rad51 complex and leads to radiation hypersensitivity and loss of G(2)/M checkpoint control. *J. Biol. Chem.* 274 32931–32935. 10.1074/jbc.274.46.32931 10551859

[B15] Chung G. Rose A. M. Petalcorin M. I. Martin J. S. Kessler Z. Sanchez-Pulido L. (2015). REC-1 and HIM-5 distribute meiotic crossovers and function redundantly in meiotic double-strand break formation in *Caenorhabditis elegans*. *Genes Dev.* 29 1969–1979. 10.1101/gad.266056.115 26385965 PMC4579353

[B16] Cressman V. L. Backlund D. C. Avrutskaya A. V. Leadon S. A. Godfrey V. Koller B. H. (1999). Growth retardation, DNA repair defects, and lack of spermatogenesis in BRCA1-deficient mice. *Mol. Cell Biol.* 19 7061–7075. 10.1128/mcb.19.10.7061 10490643 PMC84701

[B17] Cruz-Garcia A. Lopez-Saavedra A. Huertas P. (2014). BRCA1 accelerates CtIP-mediated DNA-end resection. *Cell Rep.* 9 451–459. 10.1016/j.celrep.2014.08.076 25310973

[B18] Daley J. M. Sung P. (2014). 53BP1, BRCA1, and the choice between recombination and end joining at DNA double-strand breaks. *Mol. Cell Biol.* 34 1380–1388. 10.1128/mcb.01639-13 24469398 PMC3993578

[B19] Davies O. R. Pellegrini L. (2007). Interaction with the BRCA2 C terminus protects RAD51-DNA filaments from disassembly by BRC repeats. *Nat. Struct. Mol. Biol.* 14 475–483. 10.1038/nsmb1251 17515903 PMC2096194

[B20] Deng C. X. (2002). Roles of BRCA1 in centrosome duplication. *Oncogene* 21 6222–6227. 10.1038/sj.onc.1205713 12214252

[B21] Deng C. X. (2006). BRCA1: cell cycle checkpoint, genetic instability, DNA damage response and cancer evolution. *Nucleic Acids Res.* 34 1416–1426. 10.1093/nar/gkl010 16522651 PMC1390683

[B22] Densham R. M. Garvin A. J. Stone H. R. Strachan J. Baldock R. A. Daza-Martin M. (2016). Human BRCA1-BARD1 ubiquitin ligase activity counteracts chromatin barriers to DNA resection. *Nat. Struct. Mol. Biol.* 23 647–655. 10.1038/nsmb.3236 27239795 PMC6522385

[B23] Dernburg A. F. Mcdonald K. Moulder G. Barstead R. Dresser M. Villeneuve A. M. (1998). Meiotic recombination in *C. elegans* initiates by a conserved mechanism and is dispensable for homologous chromosome synapsis. *Cell* 94 387–398. 10.1016/s0092-8674(00)81481-6 9708740

[B24] Deshaies R. J. Joazeiro C. A. (2009). RING domain E3 ubiquitin ligases. *Annu. Rev. Biochem.* 78 399–434. 10.1146/annurev.biochem.78.101807.093809 19489725

[B25] Dray E. Siaud N. Dubois E. Doutriaux M. P. (2006). Interaction between *Arabidopsis* Brca2 and its partners Rad51, Dmc1, and Dss1. *Plant Physiol.* 140 1059–1069. 10.1104/pp.105.075838 16415210 PMC1400560

[B26] Esashi F. Christ N. Gannon J. Liu Y. Hunt T. Jasin M. (2005). CDK-dependent phosphorylation of BRCA2 as a regulatory mechanism for recombinational repair. *Nature* 434 598–604. 10.1038/nature03404 15800615

[B27] Esashi F. Galkin V. E. Yu X. Egelman E. H. West S. C. (2007). Stabilization of RAD51 nucleoprotein filaments by the C-terminal region of BRCA2. *Nat. Struct. Mol. Biol.* 14 468–474. 10.1038/nsmb1245 17515904

[B28] Fernandez-Capetillo O. Mahadevaiah S. K. Celeste A. Romanienko P. J. Camerini-Otero R. D. Bonner W. M. (2003). H2AX is required for chromatin remodeling and inactivation of sex chromosomes in male mouse meiosis. *Dev. Cell* 4 497–508. 10.1016/s1534-5807(03)00093-5 12689589

[B29] Fox D. III Le Trong I. Rajagopal P. Brzovic P. S. Stenkamp R. E. Klevit R. E. (2008). Crystal structure of the BARD1 ankyrin repeat domain and its functional consequences. *J. Biol. Chem.* 283 21179–21186. 10.1074/jbc.m802333200 18480049 PMC2475683

[B30] Futreal P. A. Liu Q. Shattuck-Eidens D. Cochran C. Harshman K. Tavtigian S. (1994). BRCA1 mutations in primary breast and ovarian carcinomas. *Science* 266 120–122. 10.1126/science.7939630 7939630

[B31] Garcia V. Phelps S. E. Gray S. Neale M. J. (2011). Bidirectional resection of DNA double-strand breaks by Mre11 and Exo1. *Nature* 479 241–244. 10.1038/nature10515 22002605 PMC3214165

[B32] Garcia-Muse T. Galindo-Diaz U. Garcia-Rubio M. Martin J. S. Polanowska J. O’reilly N. (2019). A meiotic checkpoint alters repair partner bias to permit inter-sister repair of persistent DSBs. *Cell Rep.* 26 775.e5–787.e5. 30650366 10.1016/j.celrep.2018.12.074 PMC6334227

[B33] Ghimenti C. Sensi E. Presciuttini S. Brunetti I. M. Conte P. Bevilacqua G. (2002). Germline mutations of the BRCA1-associated ring domain (BARD1) gene in breast and breast/ovarian families negative for BRCA1 and BRCA2 alterations. *Genes Chromosomes Cancer* 33 235–242. 10.1002/gcc.1223 11807980

[B34] Godwin A. K. Vanderveer L. Schultz D. C. Lynch H. T. Altomare D. A. Buetow K. H. (1994). A common region of deletion on chromosome 17q in both sporadic and familial epithelial ovarian tumors distal to BRCA1. *Am. J. Hum. Genet.* 55 666–677. 7942844 PMC1918278

[B35] Gowen L. C. Johnson B. L. Latour A. M. Sulik K. K. Koller B. H. (1996). Brca1 deficiency results in early embryonic lethality characterized by neuroepithelial abnormalities. *Nat. Genet.* 12 191–194. 10.1038/ng0296-191 8563759

[B36] Gray S. Cohen P. E. (2016). Control of meiotic crossovers: from double-strand break formation to designation. *Annu. Rev. Genet.* 50 175–210. 10.1146/annurev-genet-120215-035111 27648641 PMC5319444

[B37] Gudmundsdottir K. Ashworth A. (2004). BRCA2 in meiosis: turning over a new leaf. *Trends Cell Biol.* 14 401–404. 10.1016/j.tcb.2004.07.002 15308204

[B38] Hakem R. De La Pompa J. L. Sirard C. Mo R. Woo M. Hakem A. (1996). The tumor suppressor gene Brca1 is required for embryonic cellular proliferation in the mouse. *Cell* 85 1009–1023. 10.1016/s0092-8674(00)81302-1 8674108

[B39] Hall J. M. Lee M. K. Newman B. Morrow J. E. Anderson L. A. Huey B. (1990). Linkage of early-onset familial breast cancer to chromosome 17q21. *Science* 250 1684–1689. 10.1126/science.2270482 2270482

[B40] Hartley C. L. McCulloch R. (2008). Trypanosoma brucei BRCA2 acts in antigenic variation and has undergone a recent expansion in BRC repeat number that is important during homologous recombination. *Mol. Microbiol.* 68 1237–1251. 10.1111/j.1365-2958.2008.06230.x 18430140 PMC2408642

[B41] Hashizume R. Fukuda M. Maeda I. Nishikawa H. Oyake D. Yabuki Y. (2001). The RING heterodimer BRCA1-BARD1 is a ubiquitin ligase inactivated by a breast cancer-derived mutation. *J. Biol. Chem.* 276 14537–14540. 10.1074/jbc.c000881200 11278247

[B42] Hatchi E. Skourti-Stathaki K. Ventz S. Pinello L. Yen A. Kamieniarz-Gdula K. (2015). BRCA1 recruitment to transcriptional pause sites is required for R-loop-driven DNA damage repair. *Mol. Cell* 57 636–647. 10.1016/j.molcel.2015.01.011 25699710 PMC4351672

[B43] Hill S. J. Rolland T. Adelmant G. Xia X. Owen M. S. Dricot A. (2014). Systematic screening reveals a role for BRCA1 in the response to transcription-associated DNA damage. *Genes Dev.* 28 1957–1975. 10.1101/gad.241620.114 25184681 PMC4197947

[B44] Hillers K. J. Jantsch V. Martinez-Perez E. Yanowitz J. L. (2017). Meiosis. *WormBook* 2017 1–43. 10.1016/b978-0-12-503365-7.50005-5 PMC5215044 26694509

[B45] Hong Y. Sonneville R. Agostinho A. Meier B. Wang B. Blow J. J. (2016). The SMC-5/6 complex and the HIM-6 (BLM) helicase synergistically promote meiotic recombination intermediate processing and chromosome maturation during *Caenorhabditis elegans* Meiosis. *PLoS Genet.* 12:e1005872. 10.1371/journal.pgen.1005872 27010650 PMC4807058

[B46] Hosoya N. Okajima M. Kinomura A. Fujii Y. Hiyama T. Sun J. (2011). Synaptonemal complex protein SYCP3 impairs mitotic recombination by interfering with BRCA2. *EMBO Rep.* 13 44–51. 10.1038/embor.2011.221 22116401 PMC3246250

[B47] Huber L. J. Yang T. W. Sarkisian C. J. Master S. R. Deng C. X. Chodosh L. A. (2001). Impaired DNA damage response in cells expressing an exon 11-deleted murine Brca1 variant that localizes to nuclear foci. *Mol. Cell Biol.* 21 4005–4015. 10.1128/mcb.21.12.4005-4015.2001 11359908 PMC87063

[B48] Jagut M. Hamminger P. Woglar A. Millonigg S. Paulin L. Mikl M. (2016). Separable roles for a *Caenorhabditis elegans* RMI1 homolog in promoting and antagonizing meiotic crossovers ensure faithful chromosome inheritance. *PLoS Biol.* 14:e1002412. 10.1371/journal.pbio.1002412 27011106 PMC4807110

[B49] Janisiw E. Dello Stritto M. R. Jantsch V. Silva N. (2018). BRCA1-BARD1 associate with the synaptonemal complex and pro-crossover factors and influence RAD-51 dynamics during *Caenorhabditis elegans* meiosis. *PLoS Genet.* 14:e1007653. 10.1371/journal.pgen.1007653 30383754 PMC6211622

[B50] Jensen R. B. Carreira A. Kowalczykowski S. C. (2010). Purified human BRCA2 stimulates RAD51-mediated recombination. *Nature* 467 678–683. 10.1038/nature09399 20729832 PMC2952063

[B51] Kamp J. A. Van Schendel R. Dilweg I. W. Tijsterman M. (2020). BRCA1-associated structural variations are a consequence of polymerase theta-mediated end-joining. *Nat. Commun.* 11:3615. 10.1038/s41467-020-17455-3 PMC7368036 32680986

[B52] King M. C. Marks J. H. Mandell J. B. New York Breast Cancer Study Group (2003). Breast and ovarian cancer risks due to inherited mutations in BRCA1 and BRCA2. *Science* 302 643–646. 10.1126/science.1088759 14576434

[B53] Klovstad M. Abdu U. Schupbach T. (2008). Drosophila brca2 is required for mitotic and meiotic DNA repair and efficient activation of the meiotic recombination checkpoint. *PLoS Genet.* 4:e31. 10.1371/journal.pgen.0040031 18266476 PMC2233675

[B54] Kojic M. Kostrub C. F. Buchman A. R. Holloman W. K. (2002). BRCA2 homolog required for proficiency in DNA repair, recombination, and genome stability in *Ustilago maydis*. *Mol. Cell* 10 683–691. 10.1016/s1097-2765(02)00632-9 12408834

[B55] Kojic M. Zhou Q. Lisby M. Holloman W. K. (2005). Brh2-Dss1 interplay enables properly controlled recombination in *Ustilago maydis*. *Mol. Cell Biol.* 25 2547–2557. 10.1128/mcb.25.7.2547-2557.2005 15767662 PMC1061653

[B56] Kouznetsova A. Wang H. Bellani M. Camerini-Otero R. D. Jessberger R. Hoog C. (2009). BRCA1-mediated chromatin silencing is limited to oocytes with a small number of asynapsed chromosomes. *J. Cell Sci.* 122 2446–2452. 10.1242/jcs.049353 19531582

[B57] Kowalczykowski S. C. (2015). An overview of the molecular mechanisms of recombinational DNA repair. *Cold Spring Harb. Perspect. Biol.* 7:a016410. 10.1101/cshperspect.a016410 26525148 PMC4632670

[B58] Lam I. Keeney S. (2014). Mechanism and regulation of meiotic recombination initiation. *Cold Spring Harb. Perspect. Biol.* 7:a016634. 10.1101/cshperspect.a016634 25324213 PMC4292169

[B59] Lambertini M. Goldrat O. Ferreira A. R. Dechene J. Azim H. A. Jr. Desir J. (2018). Reproductive potential and performance of fertility preservation strategies in BRCA-mutated breast cancer patients. *Ann. Oncol.* 29 237–243. 10.1093/annonc/mdx639 29045555

[B60] Leon-Ortiz A. M. Panier S. Sarek G. Vannier J. B. Patel H. Campbell P. J. (2018). A distinct class of genome rearrangements driven by heterologous recombination. *Mol. Cell* 69 292.e6–305.e6. 29351848 10.1016/j.molcel.2017.12.014 PMC5783719

[B61] Li J. Zou C. Bai Y. Wazer D. E. Band V. Gao Q. (2006). DSS1 is required for the stability of BRCA2. *Oncogene* 25 1186–1194. 10.1038/sj.onc.1209153 16205630

[B62] Li M. L. Greenberg R. A. (2012). Links between genome integrity and BRCA1 tumor suppression. *Trends Biochem. Sci.* 37 418–424. 10.1016/j.tibs.2012.06.007 22836122 PMC3459146

[B63] Li Q. Hariri S. Engebrecht J. (2020). Meiotic double-strand break processing and crossover patterning are regulated in a sex-specific manner by BRCA1-BARD1 in *Caenorhabditis elegans*. *Genetics* 216 359–379. 10.1534/genetics.120.303292 32796008 PMC7536853

[B64] Li Q. Saito T. T. Martinez-Garcia M. Deshong A. J. Nadarajan S. Lawrence K. S. (2018). The tumor suppressor BRCA1-BARD1 complex localizes to the synaptonemal complex and regulates recombination under meiotic dysfunction in *Caenorhabditis elegans*. *PLoS Genet.* 14:e1007701. 10.1371/journal.pgen.1007701 30383767 PMC6211623

[B65] Lin W. Titus S. Moy F. Ginsburg E. S. Oktay K. (2017). Ovarian aging in women with BRCA germline mutations. *J. Clin. Endocrinol. Metab.* 102 3839–3847. 10.1210/jc.2017-00765 28938488 PMC5630253

[B66] Liu C. Y. Flesken-Nikitin A. Li S. Zeng Y. Lee W. H. (1996). Inactivation of the mouse Brca1 gene leads to failure in the morphogenesis of the egg cylinder in early postimplantation development. *Genes Dev.* 10 1835–1843. 10.1101/gad.10.14.1835 8698242

[B67] Liu J. Doty T. Gibson B. Heyer W. D. (2010). Human BRCA2 protein promotes RAD51 filament formation on RPA-covered single-stranded DNA. *Nat. Struct. Mol. Biol.* 17 1260–1262. 10.1038/nsmb.1904 20729859 PMC2952495

[B68] Ludwig T. Chapman D. L. Papaioannou V. E. Efstratiadis A. (1997). Targeted mutations of breast cancer susceptibility gene homologs in mice: lethal phenotypes of Brca1, Brca2, Brca1/Brca2, Brca1/p53, and Brca2/p53 nullizygous embryos. *Genes Dev.* 11 1226–1241. 10.1101/gad.11.10.1226 9171368

[B69] Lui D. Y. Colaiacovo M. P. (2013). Meiotic development *in Caenorhabditis elegans*. *Adv. Exp. Med. Biol.* 757 133–170. 10.1007/978-1-4614-4015-4_6 22872477 PMC3764601

[B70] Mahadevaiah S. K. Bourc’his D. De Rooij D. G. Bestor T. H. Turner J. M. Burgoyne P. S. (2008). Extensive meiotic asynapsis in mice antagonises meiotic silencing of unsynapsed chromatin and consequently disrupts meiotic sex chromosome inactivation. *J. Cell Biol.* 182 263–276. 10.1083/jcb.200710195 18663141 PMC2483523

[B71] Manke I. A. Lowery D. M. Nguyen A. Yaffe M. B. (2003). BRCT repeats as phosphopeptide-binding modules involved in protein targeting. *Science* 302 636–639. 10.1126/science.1088877 14576432

[B72] Martin J. S. Winkelmann N. Petalcorin M. I. Mcilwraith M. J. Boulton S. J. (2005). RAD-51-dependent and -independent roles of a *Caenorhabditis elegans* BRCA2-related protein during DNA double-strand break repair. *Mol. Cell Biol.* 25 3127–3139. 10.1128/mcb.25.8.3127-3139.2005 15798199 PMC1069622

[B73] Martinez J. S. Von Nicolai C. Kim T. Ehlen A. Mazin A. V. Kowalczykowski S. C. (2016). BRCA2 regulates DMC1-mediated recombination through the BRC repeats. *Proc. Natl. Acad. Sci. U.S.A.* 113 3515–3520. 10.1073/pnas.1601691113 26976601 PMC4822569

[B74] McCarthy E. E. Celebi J. T. Baer R. Ludwig T. (2003). Loss of Bard1, the heterodimeric partner of the Brca1 tumor suppressor, results in early embryonic lethality and chromosomal instability. *Mol. Cell Biol.* 23 5056–5063. 10.1128/mcb.23.14.5056-5063.2003 12832489 PMC162231

[B75] Meneely P. M. Mcgovern O. L. Heinis F. I. Yanowitz J. L. (2012). Crossover distribution and frequency are regulated by him-5 in *Caenorhabditis elegans*. *Genetics* 190 1251–1266. 10.1534/genetics.111.137463 22267496 PMC3316641

[B76] Meza J. E. Brzovic P. S. King M. C. Klevit R. E. (1999). Mapping the functional domains of BRCA1. Interaction of the ring finger domains of BRCA1 and BARD1. *J. Biol. Chem.* 274 5659–5665. 10026184 10.1074/jbc.274.9.5659

[B77] Miki Y. Swensen J. Shattuck-Eidens D. Futreal P. A. Harshman K. Tavtigian S. (1994). A strong candidate for the breast and ovarian cancer susceptibility gene BRCA1. *Science* 266 66–71. 10.1126/science.7545954 7545954

[B78] Moynahan M. E. Chiu J. W. Koller B. H. Jasin M. (1999). Brca1 controls homology-directed DNA repair. *Mol. Cell* 4 511–518. 10.1016/s1097-2765(00)80202-6 10549283

[B79] Moynahan M. E. Pierce A. J. Jasin M. (2001). BRCA2 is required for homology-directed repair of chromosomal breaks. *Mol. Cell* 7 263–272. 10.1016/s1097-2765(01)00174-5 11239455

[B80] Nakamura K. Saredi G. Becker J. R. Foster B. M. Nguyen N. V. Beyer T. E. (2019). H4K20me0 recognition by BRCA1-BARD1 directs homologous recombination to sister chromatids. *Nat. Cell Biol.* 21 311–318. 10.1038/s41556-019-0282-9 30804502 PMC6420097

[B81] Neale M. J. Keeney S. (2006). Clarifying the mechanics of DNA strand exchange in meiotic recombination. *Nature* 442 153–158. 10.1038/nature04885 16838012 PMC5607947

[B82] Negrini S. Gorgoulis V. G. Halazonetis T. D. (2010). Genomic instability–an evolving hallmark of cancer. *Nat. Rev. Mol. Cell Biol.* 11 220–228. 10.1038/nrm2858 20177397

[B83] Oktay K. Kim J. Y. Barad D. Babayev S. N. (2010). Association of BRCA1 mutations with occult primary ovarian insufficiency: a possible explanation for the link between infertility and breast/ovarian cancer risks. *J. Clin. Oncol.* 28 240–244. 10.1200/jco.2009.24.2057 19996028 PMC3040011

[B84] Paiano J. Wu W. Yamada S. Sciascia N. Callen E. Paola Cotrim A. (2020). ATM and PRDM9 regulate SPO11-bound recombination intermediates during meiosis. *Nat. Commun.* 11:857. 10.1038/s41467-020-14654-w PMC7016097 32051414

[B85] Petalcorin M. I. Galkin V. E. Yu X. Egelman E. H. Boulton S. J. (2007). Stabilization of RAD-51-DNA filaments via an interaction domain in *Caenorhabditis elegans* BRCA2. *Proc. Natl. Acad. Sci. U.S.A.* 104 8299–8304. 10.1073/pnas.0702805104 17483448 PMC1895944

[B86] Petalcorin M. I. Sandall J. Wigley D. B. Boulton S. J. (2006). CeBRC-2 stimulates D-loop formation by RAD-51 and promotes DNA single-strand annealing. *J Mol. Biol.* 361 231–242. 10.1016/j.jmb.2006.06.020 16843491

[B87] Polanowska J. Martin J. S. Garcia-Muse T. Petalcorin M. I. Boulton S. J. (2006). A conserved pathway to activate BRCA1-dependent ubiquitylation at DNA damage sites. *EMBO J.* 25 2178–2188. 10.1038/sj.emboj.7601102 16628214 PMC1462971

[B88] Prakash R. Zhang Y. Feng W. Jasin M. (2015). Homologous recombination and human health: the roles of BRCA1, BRCA2, and associated proteins. *Cold Spring Harb. Perspect. Biol.* 7:a016600. 10.1101/cshperspect.a016600 25833843 PMC4382744

[B89] Reidt W. Wurz R. Wanieck K. Chu H. H. Puchta H. (2006). A homologue of the breast cancer-associated gene BARD1 is involved in DNA repair in plants. *EMBO J.* 25 4326–4337. 10.1038/sj.emboj.7601313 16957774 PMC1570427

[B90] Rodriguez M. Yu X. Chen J. Songyang Z. (2003). Phosphopeptide binding specificities of BRCA1 COOH-terminal (BRCT) domains. *J. Biol. Chem.* 278 52914–52918. 10.1074/jbc.c300407200 14578343

[B91] Roy R. Chun J. Powell S. N. (2011). BRCA1 and BRCA2: different roles in a common pathway of genome protection. *Nat. Rev. Cancer* 12 68–78. 10.1038/nrc3181 22193408 PMC4972490

[B92] Saito T. T. Colaiacovo M. P. (2017). Regulation of crossover frequency and distribution during meiotic recombination. *Cold Spring Harb. Symp. Quant. Biol.* 82 223–234. 10.1101/sqb.2017.82.034132 29222342 PMC6542265

[B93] Saito T. T. Lui D. Y. Kim H. M. Meyer K. Colaiacovo M. P. (2013). Interplay between structure-specific endonucleases for crossover control during *Caenorhabditis elegans* meiosis. *PLoS Genet.* 9:e1003586. 10.1371/journal.pgen.1003586 23874210 PMC3715419

[B94] Saito T. T. Mohideen F. Meyer K. Harper J. W. Colaiacovo M. P. (2012). SLX-1 is required for maintaining genomic integrity and promoting meiotic noncrossovers in the *Caenorhabditis elegans* germline. *PLoS Genet.* 8:e1002888. 10.1371/journal.pgen.1002888 22927825 PMC3426554

[B95] Scully R. Chen J. Plug A. Xiao Y. Weaver D. Feunteun J. (1997). Association of BRCA1 with Rad51 in mitotic and meiotic cells. *Cell* 88 265–275. 10.1016/s0092-8674(00)81847-4 9008167

[B96] Sharan S. K. Morimatsu M. Albrecht U. Lim D. S. Regel E. Dinh C. (1997). Embryonic lethality and radiation hypersensitivity mediated by Rad51 in mice lacking Brca2. *Nature* 386 804–810. 10.1038/386804a0 9126738

[B97] Sharan S. K. Pyle A. Coppola V. Babus J. Swaminathan S. Benedict J. (2004). BRCA2 deficiency in mice leads to meiotic impairment and infertility. *Development* 131 131–142. 10.1242/dev.00888 14660434

[B98] Shinohara A. Shinohara M. (2004). Roles of RecA homologues Rad51 and Dmc1 during meiotic recombination. *Cytogenet. Genome Res.* 107 201–207. 10.1159/000080598 15467365

[B99] Shivji M. K. Mukund S. R. Rajendra E. Chen S. Short J. M. Savill J. (2009). The BRC repeats of human BRCA2 differentially regulate RAD51 binding on single- versus double-stranded DNA to stimulate strand exchange. *Proc. Natl. Acad. Sci. U.S.A.* 106 13254–13259. 10.1073/pnas.0906208106 19628690 PMC2714763

[B100] Siaud N. Barbera M. A. Egashira A. Lam I. Christ N. Schlacher K. (2011). Plasticity of BRCA2 function in homologous recombination: genetic interactions of the PALB2 and DNA binding domains. *PLoS Genet.* 7:e1002409. 10.1371/journal.pgen.1002409 22194698 PMC3240595

[B101] Siaud N. Dray E. Gy I. Gerard E. Takvorian N. Doutriaux M. P. (2004). Brca2 is involved in meiosis in *Arabidopsis thaliana* as suggested by its interaction with Dmc1. *EMBO J.* 23 1392–1401. 10.1038/sj.emboj.7600146 15014444 PMC381417

[B102] Simhadri S. Peterson S. Patel D. S. Huo Y. Cai H. Bowman-Colin C. (2014). Male fertility defect associated with disrupted BRCA1-PALB2 interaction in mice. *J. Biol. Chem.* 289 24617–24629. 10.1074/jbc.m114.566141 25016020 PMC4148885

[B103] Steinfeld J. B. Belan O. Kwon Y. Terakawa T. Al-Zain A. Smith M. J. (2019). Defining the influence of Rad51 and Dmc1 lineage-specific amino acids on genetic recombination. *Genes Dev.* 33 1191–1207. 10.1101/gad.328062.119 31371435 PMC6719624

[B104] Takemoto K. Tani N. Takada-Horisawa Y. Fujimura S. Tanno N. Yamane M. (2020). Meiosis-Specific C19orf57/4930432K21Rik/BRME1 Modulates Localization of RAD51 and DMC1 to DSBs in mouse meiotic recombination. *Cell Rep.* 31:107686. 10.1016/j.celrep.2020.107686 32460033

[B105] Thai T. H. Du F. Tsan J. T. Jin Y. Phung A. Spillman M. A. (1998). Mutations in the BRCA1-associated RING domain (BARD1) gene in primary breast, ovarian and uterine cancers. *Hum. Mol. Genet.* 7 195–202. 10.1093/hmg/7.2.195 9425226

[B106] Thakur S. Zhang H. B. Peng Y. Le H. Carroll B. Ward T. (1997). Localization of BRCA1 and a splice variant identifies the nuclear localization signal. *Mol. Cell Biol.* 17 444–452. 10.1128/mcb.17.1.444 8972225 PMC231769

[B107] Thorslund T. Esashi F. West S. C. (2007). Interactions between human BRCA2 protein and the meiosis-specific recombinase DMC1. *EMBO J.* 26 2915–2922. 10.1038/sj.emboj.7601739 17541404 PMC1894777

[B108] Thorslund T. Mcilwraith M. J. Compton S. A. Lekomtsev S. Petronczki M. Griffith J. D. (2010). The breast cancer tumor suppressor BRCA2 promotes the specific targeting of RAD51 to single-stranded DNA. *Nat. Struct. Mol. Biol.* 17 1263–1265. 10.1038/nsmb.1905 20729858 PMC4041013

[B109] Turner J. M. (2007). Meiotic sex chromosome inactivation. *Development* 134 1823–1831. 10.1242/dev.000018 17329371

[B110] Turner J. M. Aprelikova O. Xu X. Wang R. Kim S. Chandramouli G. V. (2004). BRCA1, histone H2AX phosphorylation, and male meiotic sex chromosome inactivation. *Curr. Biol.* 14 2135–2142. 10.1016/j.cub.2004.11.032 15589157

[B111] Tutt A. Gabriel A. Bertwistle D. Connor F. Paterson H. Peacock J. (1999). Absence of Brca2 causes genome instability by chromosome breakage and loss associated with centrosome amplification. *Curr. Biol.* 9 1107–1110. 10.1016/s0960-9822(99)80479-5 10531007

[B112] Venkitaraman A. R. (2002). Cancer susceptibility and the functions of BRCA1 and BRCA2. *Cell* 108 171–182. 10.1016/s0092-8674(02)00615-3 11832208

[B113] Volkova N. V. Meier B. Gonzalez-Huici V. Bertolini S. Gonzalez S. Vohringer H. (2020). Mutational signatures are jointly shaped by DNA damage and repair. *Nat. Commun.* 11:2169. 10.1038/s41467-020-15912-7 PMC7195458 32358516

[B114] Wagner C. R. Kuervers L. Baillie D. L. Yanowitz J. L. (2010). xnd-1 regulates the global recombination landscape in *Caenorhabditis elegans*. *Nature* 467 839–843. 10.1038/nature09429 20944745 PMC3045774

[B115] Weinberg-Shukron A. Rachmiel M. Renbaum P. Gulsuner S. Walsh T. Lobel O. (2018). Essential role of BRCA2 in ovarian development and function. *N. Engl. J. Med.* 379 1042–1049. 30207912 10.1056/NEJMoa1800024 PMC6230262

[B116] Wong A. K. Pero R. Ormonde P. A. Tavtigian S. V. Bartel P. L. (1997). RAD51 interacts with the evolutionarily conserved BRC motifs in the human breast cancer susceptibility gene brca2. *J. Biol. Chem.* 272 31941–31944. 10.1074/jbc.272.51.31941 9405383

[B117] Wooster R. Bignell G. Lancaster J. Swift S. Seal S. Mangion J. (1995). Identification of the breast cancer susceptibility gene BRCA2. *Nature* 378 789–792. 8524414 10.1038/378789a0

[B118] Wu L. C. Wang Z. W. Tsan J. T. Spillman M. A. Phung A. Xu X. L. (1996). Identification of a RING protein that can interact in vivo with the BRCA1 gene product. *Nat. Genet.* 14 430–440. 10.1038/ng1296-430 8944023

[B119] Xia B. Sheng Q. Nakanishi K. Ohashi A. Wu J. Christ N. (2006). Control of BRCA2 cellular and clinical functions by a nuclear partner. PALB2. *Mol. Cell* 22 719–729. 10.1016/j.molcel.2006.05.022 16793542

[B120] Xu X. Aprelikova O. Moens P. Deng C. X. Furth P. A. (2003). Impaired meiotic DNA-damage repair and lack of crossing-over during spermatogenesis in BRCA1 full-length isoform deficient mice. *Development* 130 2001–2012. 10.1242/dev.00410 12642502

[B121] Xu X. Qiao W. Linke S. P. Cao L. Li W. M. Furth P. A. (2001). Genetic interactions between tumor suppressors Brca1 and p53 in apoptosis, cell cycle and tumorigenesis. *Nat. Genet.* 28 266–271. 10.1038/90108 11431698

[B122] Xu X. Weaver Z. Linke S. P. Li C. Gotay J. Wang X. W. (1999). Centrosome amplification and a defective G2-M cell cycle checkpoint induce genetic instability in BRCA1 exon 11 isoform-deficient cells. *Mol. Cell* 3 389–395. 10.1016/s1097-2765(00)80466-9 10198641

[B123] Yang H. Jeffrey P. D. Miller J. Kinnucan E. Sun Y. Thoma N. H. (2002). BRCA2 function in DNA binding and recombination from a BRCA2-DSS1-ssDNA structure. *Science* 297 1837–1848. 10.1126/science.297.5588.1837 12228710

[B124] Yarden R. I. Pardo-Reoyo S. Sgagias M. Cowan K. H. Brody L. C. (2002). BRCA1 regulates the G2/M checkpoint by activating Chk1 kinase upon DNA damage. *Nat. Genet.* 30 285–289. 10.1038/ng837 11836499

[B125] Yokoo R. Zawadzki K. A. Nabeshima K. Drake M. Arur S. Villeneuve A. M. (2012). COSA-1 reveals robust homeostasis and separable licensing and reinforcement steps governing meiotic crossovers. *Cell* 149 75–87. 10.1016/j.cell.2012.01.052 22464324 PMC3339199

[B126] Yu V. P. Koehler M. Steinlein C. Schmid M. Hanakahi L. A. Van Gool A. J. (2000). Gross chromosomal rearrangements and genetic exchange between nonhomologous chromosomes following BRCA2 inactivation. *Genes Dev.* 14 1400–1406. 10837032 PMC316655

[B127] Yu X. Chini C. C. He M. Mer G. Chen J. (2003). The BRCT domain is a phospho-protein binding domain. *Science* 302 639–642. 10.1126/science.1088753 14576433

[B128] Yu Z. Kim Y. Dernburg A. F. (2016). Meiotic recombination and the crossover assurance checkpoint in *Caenorhabditis elegans*. *Semin. Cell Dev. Biol.* 54 106–116. 10.1016/j.semcdb.2016.03.014 27013114 PMC5082714

[B129] Yuan S. S. Lee S. Y. Chen G. Song M. Tomlinson G. E. Lee E. Y. (1999). BRCA2 is required for ionizing radiation-induced assembly of Rad51 complex in vivo. *Cancer Res.* 59 3547–3551. 10446958

[B130] Zetka M. C. Rose A. M. (1995). Mutant rec-1 eliminates the meiotic pattern of crossing over in *Caenorhabditis elegans*. *Genetics* 141 1339–1349. 10.1093/genetics/141.4.1339 8601478 PMC1206871

[B131] Zhang J. Fujiwara Y. Yamamoto S. Shibuya H. (2019). A meiosis-specific BRCA2 binding protein recruits recombinases to DNA double-strand breaks to ensure homologous recombination. *Nat. Commun.* 10:722. 10.1038/s41467-019-08676-2 PMC6374363 30760716

[B132] Zhang J. Gurusaran M. Fujiwara Y. Zhang K. Echbarthi M. Vorontsov E. (2020). The BRCA2-MEILB2-BRME1 complex governs meiotic recombination and impairs the mitotic BRCA2-RAD51 function in cancer cells. *Nat. Commun.* 11:2055. 10.1038/s41467-020-15954-x PMC7188823 32345962

[B133] Zhao W. Steinfeld J. B. Liang F. Chen X. Maranon D. G. Jian Ma C. (2017). BRCA1-BARD1 promotes RAD51-mediated homologous DNA pairing. *Nature* 550 360–365. 10.1038/nature24060 28976962 PMC5800781

[B134] Zickler D. Kleckner N. (2015). Recombination, pairing, and synapsis of homologs during meiosis. *Cold Spring Harb. Perspect. Biol.* 7:a016626. 10.1101/cshperspect.a016626 25986558 PMC4448610

